# Determinants of exercise intolerance symptoms considered non-specific for heart failure in patients with stage A and B: role of the left atrium in the transition phase to overt heart failure

**DOI:** 10.1007/s10554-021-02375-0

**Published:** 2021-08-30

**Authors:** Caterina Maffeis, Riccardo M. Inciardi, Muhammad Shahzeb Khan, Elvin Tafciu, Corinna Bergamini, Giovanni Benfari, Martina Setti, Flavio L. Ribichini, Mariantonietta Cicoira, Javed Butler, Andrea Rossi

**Affiliations:** 1 Division of Cardiology, Department of Medicine, University Hospital of Verona, University of Verona, P.le Stefani 1, 37126 Verona, Italy; 2 Department of Medicine, University of Mississippi Medical Center, Jackson, MS USA

**Keywords:** Heart failure, Preserved left ventricular function, Left atrial strain, Atrial cardiomyopathy

## Abstract

To assess to what extent left atrial (LA) structure and function are associated with non-specific heart failure symptoms, so that patients were classified as HF stage A and B. Mechanisms underlying the transition to overt HF in patients with stage A and B HF are unclear. Consecutive outpatients undergoing echocardiography and clinical evaluation and classified as HF stage A and B with preserved left ventricular ejection fraction (LVEF) were included. The association between LA measures [volume (LAVi), peak longitudinal-(PALS), contraction-(PACS) and conduit-strain] and non-specific HF symptoms was assessed using adjusted logistic regression analyses. The incremental value of atrial myopathy in symptoms prediction on top of clinical or echocardiographic confounders was assessed through ROC curves analyses. The cohort comprehended 185 patients (63 ± 16 years, 47% women) of whom 133 (72%) were asymptomatic, and 52 (28%) reported non-specific HF symptoms. After adjustment for clinical and echocardiographic confounders for HF symptoms, LAVi, PALS and PACS were associated with symptoms (p < 0.05). Among echocardiographic variables, only LA parameters were significantly associated with symptoms on top of clinical confounders (for LAVi OR [95% CI] 1.56 [1.21–2.00], p < 0.0001; for PALS 1.45 (1.10–1.91), p = 0.0009; for PACS 2.10 [1.33–3.30], p = 0.002). After adjustment for age, hypertension and COPD or E/E′, LV mass-i and mitral ERO, atrial myopathy added predictive value for symptoms presence compared to the clinical variables or echocardiographic parameters described (AUC increase 0.80 to 0.88, p = 0.004, and 0.79 to 0.84, p = 0.06, respectively). In patients with HF stages A–B and preserved LVEF, measures of LA structure and function were associated with non-specific HF symptoms. A comprehensive LA remodeling evaluation may help clinicians in the appropriate identification of overt HF.

**Supplementary Information:**

The online version contains supplementary material available at 10.1007/s10554-021-02375-0.

## Introduction

The pathophysiological mechanisms related to the onset of dyspnea are not well known especially in elderly patients with subtle cardiac abnormalities [1]. Self-reported dyspnea has been associated with incident heart failure (HF) and mortality in the general population, representing the transition phase to overt HF [2, 3]. While dyspnea may be caused by a combination of comorbidities such as obesity, pulmonary disease, muscular disorders and cognitive impairment, dyspnea mainly represents a critical symptom of HF [3] in which left ventricular (LV) systolic and diastolic dysfunction are thought to be key contributors [4]. However, community-based studies have shown conflicting results regarding the association of LV dysfunction and symptoms, leading to underdiagnosis of HF especially among patients with preserved ejection fraction [5–7].

Emerging data show that left atrial (LA) structure and function may be independently associated with or may even precede HF onset in asymptomatic patients [8–10]. A “stiff LA syndrome” has been described as a consequence of a less compliant LA on exercise capacity despite preserved LV systolic and diastolic function [11]. Since LA plays a key role in the modulation of LV filling and function, a reduced atrial reserve may represent the first sign of a failing heart and thus mark the onset of dyspnea. Until now, majority of the studies have assessed the relationship between LA dysfunction and symptoms in patients with known cardiovascular diseases [12]. However, it remains unknown to what extent LA dysfunction may be associated with the onset of dyspnea in stage A and B HF patients.

Therefore, the aims of this study were: (1) characterizing non-specific symptoms, not referable to HF, in ambulatory patients, who were therefore classified as HF stages A or B and (2) testing the hypothesis that an impairment in LA structure and function may contribute to the presence of non-specific HF symptoms in such patients adding incremental information to the conventional non-invasive measures of cardiac structure and function and comorbidities.

## Methods

### Patients population

Consecutive patients referred to the echocardiographic laboratory of our Institute for cardiologic evaluation with clinical data available were screened. A recent ambulatory visit performed by a cardiologist was required during the prior 3 months or immediately after echocardiography. Patients included had to fulfil the following criteria: (1) HF Stage A or B defined as presence of conditions that are strongly associated with the development of HF with [stage B] or without [stage A] structural heart disease; (2) no previous history of clear signs or symptoms of HF; (3) absence of major criteria for HF at the time of the cardiologic evaluation according to Framingham criteria [13]; (4) absence of clinical diagnosis or suspicion of unstable angina; (5) echocardiographic evidence of preserved LV systolic function defined as LV ejection fraction (LVEF) > 50%; 6) sinus rhythm at the time of echocardiography. Patients with the following conditions were excluded: (1) unavailable measures of LA structure and function; (2) history of cardiac surgery or myocardial infarction in the previous 6 months; (3) any degree of mitral stenosis, rheumatic involvement of mitral valve, mitral valve prosthesis, moderate to severe aortic disease; (4) known pre-capillary pulmonary hypertension; (5) severe or active known lung disease (asthma exacerbation or advanced stage of chronic obstructive pulmonary disease [COPD]), who have required supplemental oxygen or previous hospitalization. The work has been carried out in accordance with the Declaration of Helsinki. Institutional review board approval was obtained for this project and written informed consent was obtained according to local regulation.

Clinical history and physical examination were recorded for each patient including age, sex, history of coronary artery disease (CAD), percutaneous or surgical revascularization, paroxysmal or persistent atrial fibrillation (AF), hypertension, hyperlipidemia, smoking status, COPD, asthma, chronic kidney disease (defined as GFR_MDRD_ ≤ 60 mL/min/1.73 m^2^), diabetes. When available, a venous blood samples acquired during the previous 3 months was collected.

The symptomatic status was verified by reviewing clinical reports with particular attention to the presence of perceived breathlessness in daily activities referred by the patient in the absence of any sign of pulmonary congestion, which was considered as non-specific symptom by the cardiologist and justified by age, comorbidities or deconditioning and therefore unsufficient to classify the patient as evolved to the symptomatic phase of HF. Indeed, dyspnea on exertion is a minor criterion for HF according to Framingham criteria and, alone, it doesn’t classify the patient as symptomatic for HF [13].

### 2D echocardiography

All patients underwent a comprehensive echocardiographic evaluation according to the last Echocardiographic Guidelines of American Society of Echocardiography (ASE)/European Society of Cardiology (ESC) [14]. LV end-diastolic volume (EDV) and LV end-systolic volume (ESV) were measured using the biplane Simpson method and EF was calculated. LV mass was calculated according to the ASE convention using two-dimensional echocardiography from the parasternal long-axis view: LV wall mass (gr) = 0.8 × (1 0.04((LVIDd + IVDs + PWd) 3 − (LVIDd) 3)) + 0.6 [14]. LA volume was measured at end-systole in apical-four-chamber view, by tracing the LA inner border excluding the area under the mitral valve annulus and the inlet of the pulmonary veins. Measures were indexed by body surface area (EDVi and LAVi). Lateral and septal Doppler tissue imaging-derived systolic (S-TDI) and diastolic velocities were measured, and mean E/E' ratio was calculated. The presence of diastolic dysfunction was evaluated and graded according to recommended algorithms. In presence of mitral regurgitation (MR), effective regurgitant orifice area (ERO) was calculated by means of proximal iso-velocity surface area method [15]. Pulmonary artery systolic pressure (PASP) was obtained by the following formula as recommended: 4*(peak tricuspid regurgitation velocity)^2^ + right atrial pressure.

### 2D‑speckle tracking echocardiography

For LA echocardiographic speckle-tracking analysis, 2D grayscale images were acquired in the standard apical four-chamber view at a frame rate of 50 frames/s. Three consecutive heart cycles were recorded during a quiet breath hold. The analysis was performed offline using dedicated semi-automated acoustic tracking software (QLAB9; Philips). To calculate LA longitudinal strain, the QRS onset was used as reference point. After identifying the endocardial inner region of interest composed of six segments, tracking quality analysis and eventual manual adjustment were performed. Peak atrial longitudinal strain (PALS), which corresponds to the end of the reservoir phase, was calculated by averaging the peak values observed in all LA segments. Peak atrial contraction strain (PACS) corresponds to the positive peak at the onset of the p wave, at the end of the conduit phase in late diastole. Conduit strain (CS) was derived by the difference between PALS and PACS.

In patients in whom a maximum of two out of six segments were excluded because of inadequate tracking, strains values were calculated by averaging the values measured in the remaining segments. The reproducibility and feasibility of LA speckle-tracking measurement has been previously reported by studies conducted in our echocardiographic laboratory [16]. Patients were divided according to LA enlargement (≥ 35 mL/m^2^) and/or dysfunction. LA reservoir function (PALS) with a cut-off of 38% and LA booster function (PACS) with a cut-off of 16% were used as representative of LA dysfunction [17]. Four stages of atrial myopathy were defined: 0) normal LAVi, PALS and PACS; (1) normal LAVi, reduced PALS or PACS; (2) dilated LAVi, normal PALS and PACS or one of the two reduced; (3) dilated LAVi, reduced PALS and PACS.

### Statistical analyses

Data are expressed as mean ± standard deviation, median (25th–75th percentiles) or frequencies (%). The Shapiro–Wilk test was performed to assess data normal distribution. Baseline characteristics were compared between asymptomatic and symptomatic patients, regardless of discrimination between HF stage A and B, using t-test or nonparametric test for continuous variables [18] and χ^2^ test for binary variables. Associations between symptomatic status and measures of LA structure (LAVi) and function (PALS, PACS and CS) were assessed with logistic regression analysis. After testing the unadjusted association, two models were constructed. Model 1 included age, sex, body mass index, hypertension, eGFR, history of CAD, total cholesterol, diabetes and COPD. Model 2 included S-TDI, E/E', LV mass-i and MR-ERO. A third model was constructed (Model 3), which included all the covariates considered in Model 1 and in Model 2. The covariates included in the models were a selection of variables significantly associated with symptomatic status (Tables 1 and 2) and plausible demographic and clinical confounders with symptoms. Receiver operating characteristic (ROC) curves were generated to assess the performance of individual LA structural (LAVi) and functional parameters (PALS, PACS and CS) to predict the symptomatic status. Furthermore, the presence of enlarged LA and/or reduced LA function were considered as expression of atrial myopathy and patients were divided in the previously described four stages. To assess the incremental value of atrial myopathy in symptoms prediction in comparison to clinical or echocardiographic parameters alone, ROC curves analyses and DeLong test were performed. Three covariates were selected from each of the two previously described models (Model 1 and Model 2) based on statistical significance at t-test and χ^2^ test. Two ROC curves analyses were performed, which included in addition to atrial myopathy, age, hypertension and COPD; LV mass-i, E/E′ and mitral ERO, respectively. All tests were two-tailed. A p value < 0.05 was considered to indicate statistical significance. Analyses were performed using SPSS version 20.0 (SPSS, Chicago, IL).

**Table 1:** Clinical characteristics of the study population stratified by symptomatic status

	Asymptomatic n = 133 (72%)	Symptomatic n = 52 (28%)	p value
Age (years)	58 [49,71]	75.5 [68,81]	< 0.001
Female	59 (44.4%)	20 (53.8%)	0.26
Hypertension	65 (48.9%)	44 (84.6%)	< 0.001
Diabetes	30 (22.6%)	18 (34.6%)	0.19
Dyslipidemia	53 (39.8%)	31 (59.6%)	0.09
Coronary artery disease	26 (19.5%)	14 (26.9%)	0.32
History of AF	11 (8.3%)	9 (17.3%)	0.11
History of asthma or COPD	2 (1.5%)	7 (13.5%)	0.003
Smoking	28 (21.1%)	15 (28.8%)	0.45
eGFR < 60 mL/min/1.73 m^2^	16 (12.0%)	27 (51.9%)	< 0.0001
eGFR (mL/min/1.73 m^2^)	82.5 [66.2,104.1]	54.2 [43.6,84.9]	< 0.0001
BMI, kg/m2	25.1 [22.6,27.3]	23.4 [21.5,25.6]	0.09
Total Cholesterol (mg/dL)	179 [155.5,208.5]	163.5 [131.7,207.2]	0.03
LDL cholesterol (mg/dL)	99.5 [75.5,122.2]	78 [62.2,107.5]	0.008
Blood Glucose (mg/dL)	93 [85.5,106]	95 [83.2,111.5]	0.23
Systolic blood pressure (mmHg)	130 [120,140]	130 [120,140]	0.17
Diastolic blood pressure (mmHg)	80 [70,80]	70 [70,80]	0.17

Data displayed as n (%) or median [25th,75th percentiles]

*AF* atrial fibrillation, *BMI* body mass index, *COPD* chronic obstructive pulmonary disease, *eGFR* estimated glomerular filtration rate by the Modification of Diet in Renal Disease equation, *LDL* low-density lipoprotein

**Table 2:** Echocardiographic characteristics of the study population stratified by symptomatic status

	Mean ± SD		p value	p value adjusted for model 1
	Asymptomatic n = 133 (72%)	Symptomatic n = 52 (28%)		
LV-EDVi (mL/m^2^)	62.4 ± 14.7	62.9 ± 15.0	0.8	0.8
LV-mass-i (g/m^2^)	100 ± 33	129 ± 41	< 0.0001	0.09
LVEF (%)	62.7 ± 5.4	61.0 ± 6.6	0.07	0.8
S-TDI (cm/s)	8.2 ± 1.6	7.4 ± 1.7	0.01	0.6
TAPSE (mm)	23.8 ± 3.3	23.2 ± 3.7	0.3	0.37
E/A ratio	1.1 ± 0.4	1.06 ± 0.5	0.5	0.2
E/E′	8.9 ± 3.4	11.7 ± 3.8	< 0.0001	0.4
ERO (cm/m^2^)	0.06 ± 0.12	0.10 ± 0.13	< 0.0001	0.16
PASP (mmHg)	28.9 ± 6.1	35.8 ± 12.2	< 0.0001	0.16
LAVi (mL/m^2^)	32.1 ± 12.5	64.0 ± 15.1	< 0.0001	< 0.0001
LA PALS (%)	36.0 ± 9.8	26.8 ± 11.2	< 0.0001	0.009
LA PACS (%)	16.6 ± 6.1	12.1 ± 6.3	< 0.0001	0.002
LA conduit (%)	19.6 ± 7.5	14.6 ± 6.8	< 0.0001	0.36

All listed parameters were entered into separate multivariate models

*Adjustment* Age, Sex, BMI, Hypertension, eGFR, history of coronary artery disease, total cholesterol, diabetes, COPD. *LV-EDVi* left ventricular end-diastolic volume index, *LV-mass-i* left ventricular mass/BSA, *LVEF* left ventricular ejection fraction, *DTE* deceleration time E wave, *LAVi* left atrial volume index, *LA PALS* LA peak atrial longitudinal strain, *LA PACS* LA peak atrial contraction strain, *ERO* mitral effective regurgitant orifice, *PASP* pulmonary artery systolic pressure

## Results

The cohort consisted of 185 patients [mean age 63 ± 16 years, 47% women] of whom 64 (35%) were classified as stage A HF and 121 (65%) as stage B HF. One-hundred-nine patients (59%) had hypertension, 48 (26%) diabetes and 20 (11%) history of AF. Forty patients (22%) were routinely followed for CAD, 28 (15%) for heart valve disease and 20 (11%) for exclusion of drug-induced cardiotoxicity. Ninety-six patients (57%) were evaluated due to a specific murmur, palpitation, or cardiovascular risk factors.

Overall, mean EDVi was 63 ± 15 mL, EF 62 ± 6% and S-TDI 8.0 ± 1.7 cm/s. LA was enlarged in 86 patients (46%). Mean E/E′ was 9.7 ± 3.8; E/E′ was > 14 in 16 patients (9%). Diastolic dysfunction was evaluated and graded according to recommended algorithms [19] and shown in Supplementary Table 1. Mean PALS was 33 ± 11%, PACS 15 ± 6% and CS 18 ± 7%. MR was present in 84 patients (45%) with a mean ERO of 0.07 ± 0.12 cm^2^.

Tables 1 and 2 show clinical and echocardiographic characteristics of patients according to presence of symptoms. One-hundred-thirty-three patients (72%) were asymptomatic and 52 (28%) referred breathlessness, classified as non-specific HF symptom. Symptomatic patients were more likely to be older, to suffer from hypertension, chronic kidney disease, to have higher levels of total and low-density lipoprotein cholesterol and to have history of non-severe COPD or asthma (Table 1). Overall measures of cardiac structure and function were impaired in the symptomatic group (Table 2). In particular, patients with symptoms had lower S-TDI, higher LV-mass, E/E′, MR-ERO, PASP but not lower TAPSE.

**Fig. 1:**
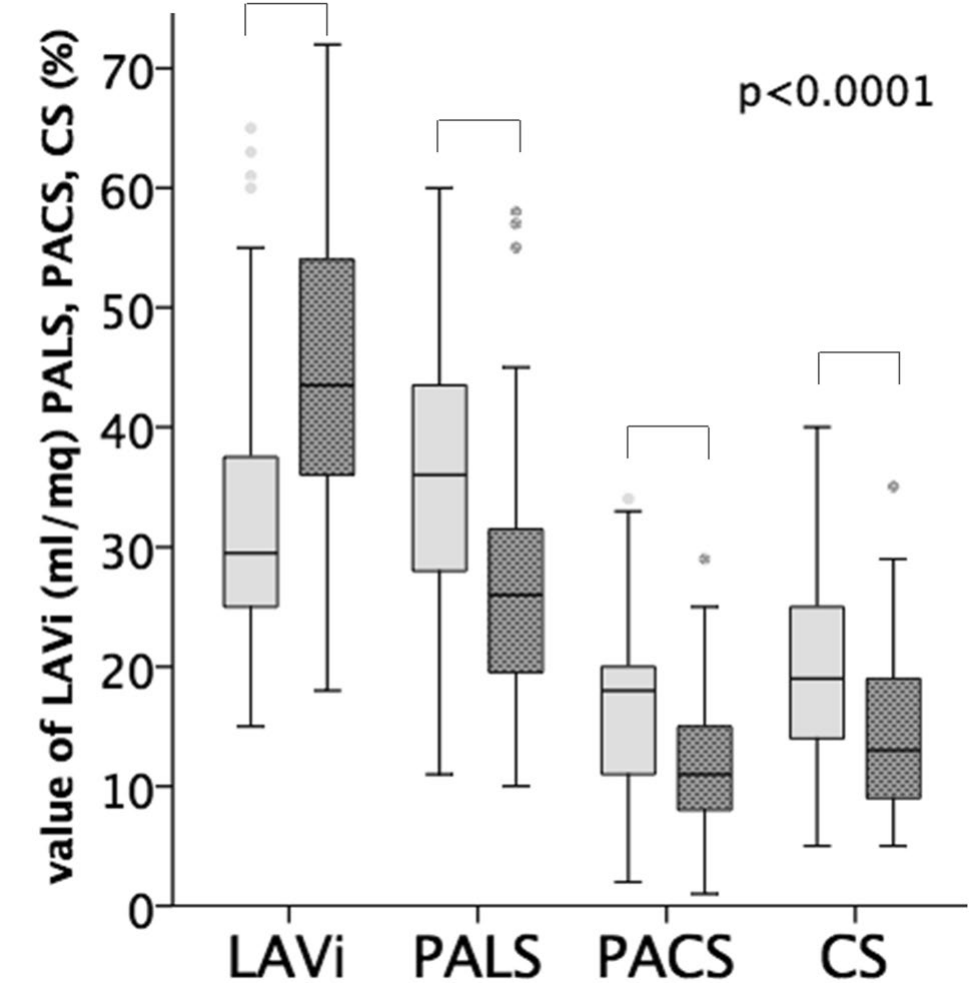
Measures of left atrial (LA) structure (left atrial volume index [LAVi]) and function (peak atrial-longitudinal strain [PALS], -contraction strain [PACS] and—conduit strain [CS]) in the asymptomatic and the symptomatic group (between groups: p < 0.0001 for all). Light grey boxes represent asymptomatic patients, dark grey boxes represent symptomatic patients

Measures of LA structure, LAVi, and function, PALS, PACS and CS, were significantly different between the two groups (Table 2; Fig. 1). A significant association between each measure of LA structure and function and symptomatic status was observed (Table 3). LAVi, PALS and PACS, but not CS, remained significantly associated with symptomatic status after adjustment for clinical and echocardiographic confounders (Table 3). Moreover, only LAVi, PALS and PACS, among all echocardiographic variables, had independent association with symptoms after comprehensive adjustment for clinical variables included in Model 1 (Table 2). LAVi was the most accurate in predicting symptoms (AUC:0.81) followed by PALS (AUC:0.75), E/E′ (AUC: 0.73) and PACS (AUC: 0.71).

**Table 3:** Association between Symptomatic Status and LA structure and function

	Unadjusted		Model 1		Model 2		Model 3	
	OR, 95% CI	p value	OR, 95% CI	p value	OR, 95% CI	p value	OR, 95% CI	p value
LAVi (mL/m^2^)	1.43 (1.24–1.64)	< 0.0001	1.56 (1.21–2.00)	< 0.0001	1.32 (1.06–1.65)	0.01	1.48 (1.01–2.17)	0.04
LA PALS (%)	1.59 (1.31–1.93)	< 0.0001	1.45 (1.10–1.91)	0.009	1.29 (1.01–1.64)	0.03	1.64 (1.03–2.65)	0.03
LA PACS (%)	1.82 (1.36–2.45)	< 0.0001	2.10 (1.33–3.30)	0.002	1.60 (1.10–2.31)	0.01	5.82 (1.87–18.1)	0.002
LA CS (%)	1.64 (1.27–2.13)	< 0.0001	1.12 (0.80–1.76)	0.3	1.09 (0.78–1.51)	0.6	0.91 (0.48–1.72)	0.7

*OR* Odds ratio, *CI* confidence interval, *OR* is expressed per 5-unit change in each measure of LAVi (increase) and LA function (decrease). *Model 1* adjusted for Age, Sex, Body mass index, Hypertension, eGFR, history of coronary artery disease, total cholesterol, diabetes, COPD, *Model 2* adjusted for S-TDI, LV mass-index, E/E', MR-ERO**,** *Model 3* adjusted for Age, Sex, Body mass index, Hypertension, eGFR, history of coronary artery disease, total cholesterol, diabetes, COPD, S-TDI, LV mass-index, E/E', MR-ERO**.** *LAVi* left atrial volume index, *LA PALS* LA peak atrial longitudinal strain, *LA PACS* LA peak atrial contraction strain, *LA CS* conduit strain

**Fig. 2:**
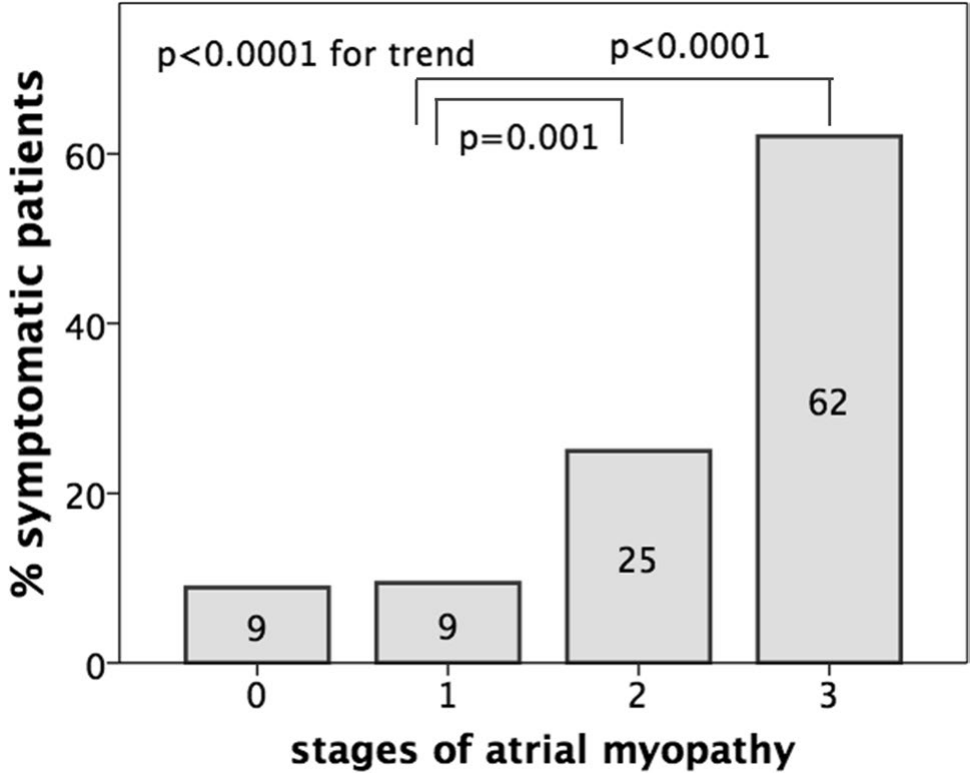
Prevalence of non-specific HF symptoms in the four stages of atrial myopathy

**Fig. 3:**
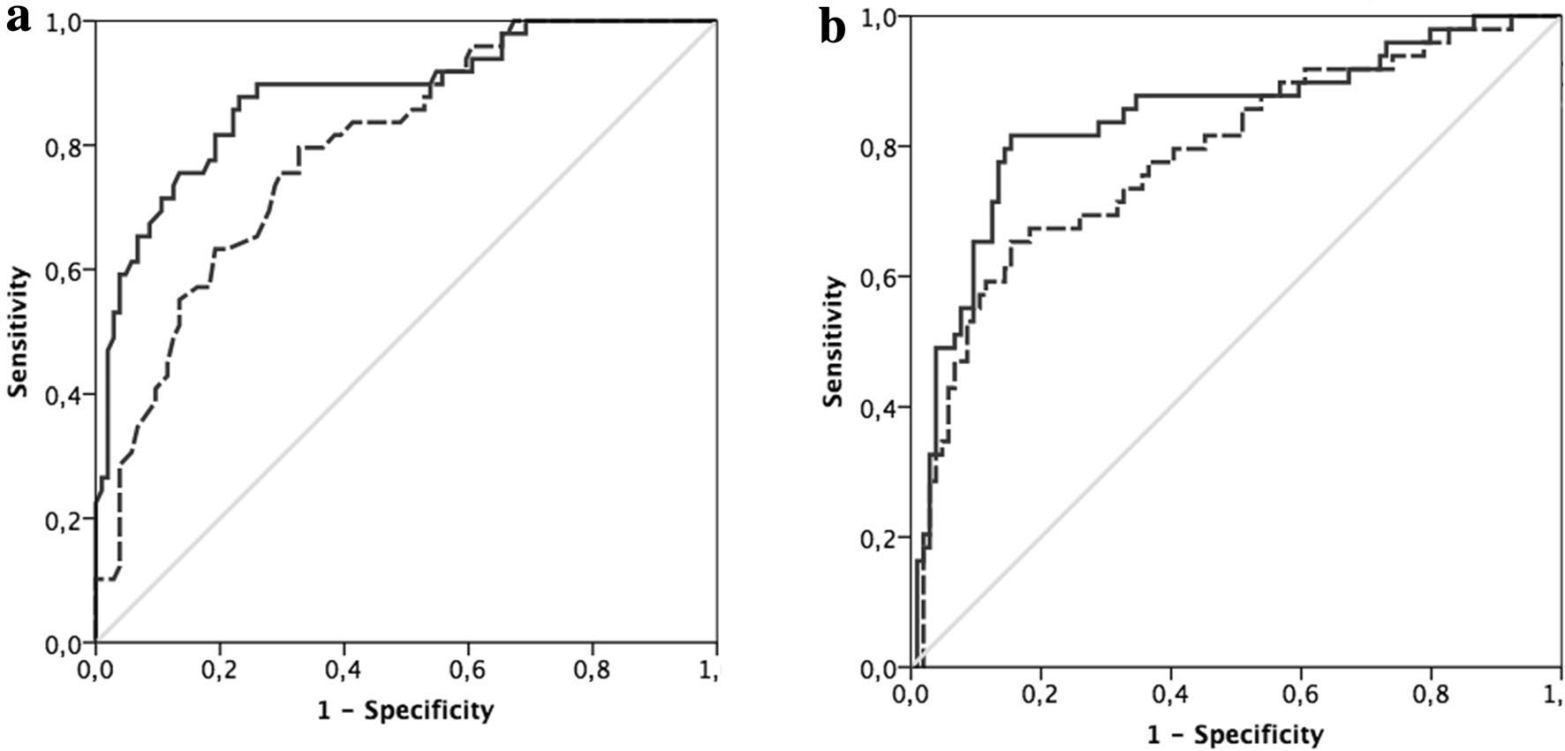
Incremental predictive value of LA myopathy. Atrial myopathy was graded as abnormal left atrial volume (LAVi) and/or peak longitudinal strain (PALS) and/or contraction strain (PACS). Notably, the addition of atrial myopathy to clinical (age, Hypertension, COPD) (**a**) or echocardiographic confounders (E/E′, LV mass-i, mitral effective regurgitant orifice area) (**b**) significantly improved the predictive value for symptoms (AUC increases from 0.80 to 0.88, p = 0.004, and from 0.79 to 0.84, p = 0.06, respectively)

Mean values of LA strain and LAVi according to normal/abnormal LAVi or PALS are reported in Supplementary Table 2. In the overall population, 46 patients (25%) had normal LAVi and normal LA function (PALS and PACS) (stage 0); 53 (29%) had reduced PALS or PACS but normal LAVi (stage 1); 28 (15%) had dilated LAVi with preserved function (PALS and PACS) or dilated LAVi with impairment in PALS or PACS (stage 2); 58 (31%) showed both structural and functional (PALS and PACS) impairment (stage 3). On comparison among the four groups, symptoms were more likely to be present in advanced stage of atrial myopathy (p for trend < 0.0001) (Supplementary Table 3; Fig. 2; Supplementary Fig. 1). The incremental value of the presence of atrial myopathy in symptoms prediction on top of clinical and echocardiographic confounders was demonstrated using ROC curves analyses (Fig. 3a, b). The clinical model included age, hypertension and COPD; the echocardiographic model included E/E′, LV mass-i and mitral ERO. The further addition of atrial myopathy added predictive value to the models with AUC increase from 0.80 to 0.88 (p = 0.004) and from 0.79 to 0.84 (p = 0.06), respectively (Fig. 3a, b).

Results remained similar in the subgroup of patients without history of AF (n = 165) [Supplementary Table 4].

## Discussion

**Fig. 4:**
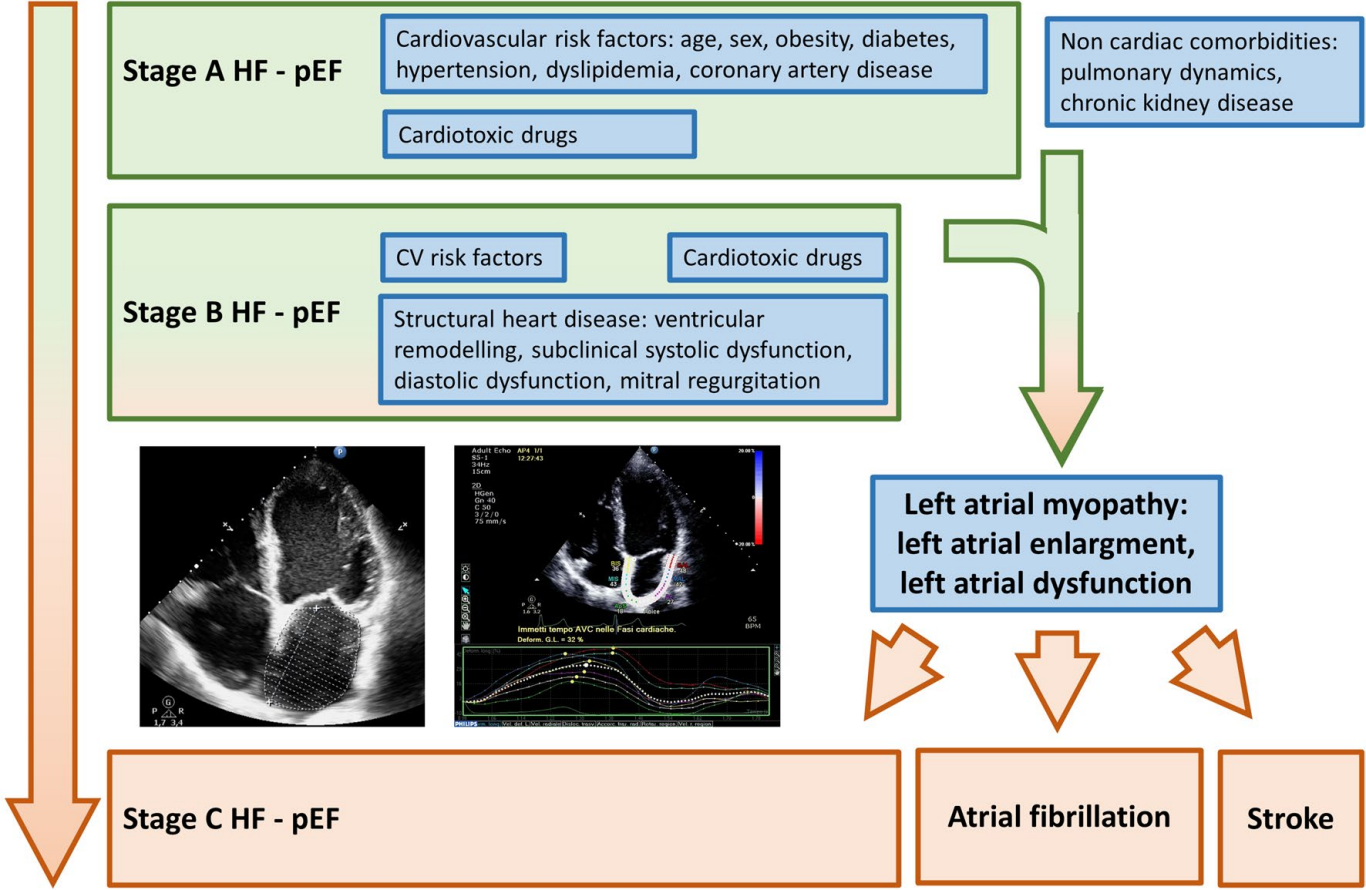
Central role of left atrial myopathy in the transition from the asymptomatic (stages A–B) to the symptomatic phase (stage C) of heart failure with preserved left ventricular ejection fraction (HF-pEF)

We report several key findings in this study. First, in patients with HF stages A–B and preserved LVEF, measures of LA structure and function are the only echocardiographic parameters associated with non-specific referred symptoms independently of non-cardiac comorbidities. Second, LA measures are also significantly associated with symptoms after accounting for echocardiographic parameters that are strongly linked with the development of HF such as LV mass, indices of systolic and diastolic function, quantitatively graded mitral regurgitation. Third, LA myopathy evaluation through structural (LAVi) and functional parameters (PALS and PACS) provides incremental information in identifying symptomatic status of patients after assessment of comorbidities and structural pre-requisites for the development of HF (Fig. 4).

We found that measures of LA remodeling are significantly associated with non-specific symptoms showing a significant discrimination accuracy compared with other cardiac measures. The LA is commonly considered a buffer chamber between the pulmonary circulation and the LV and its changes are indirectly thought to be related to LV function [10]. Chronic exposure to high LV filling pressure initiates an adaptive process leading to LA enlargement and dysfunction [20]. However, our data showed that in patients with preserved LVEF, LA impairment was predictive of symptoms regardless of age, sex and the common measures of LV structure and systolic/diastolic function (i.e. LV hypertrophy, S-TDI, E/E`). Thus, it is possible that, especially in the context of preserved EF, underlying LA functional impairment per se may contribute to the onset of symptoms and should not be considered just an innocent bystander [21].

LA modulates LV filling and function by the reservoir phase, when LA chamber collects blood coming from the pulmonary veins, the conduit phase, when blood passively transfers to the LV in early diastole and the contraction phase when blood is forced to fill LV at end-diastole. LA also acts as interplay between the LV and the pulmonary circulation, buffering pressure and flow oscillations during the cyclic cardiac work [11, 22]. The loss of global atrial function may directly affect LV output, especially during exercise [10]. Also, reduced LA wall compliance, which influences atrial reservoir function, results in elevated LA pressure which consequently can lead to increased pulmonary venous and arterial pressure and thus symptoms [23]. From this perspective, assessment of LA function by speckle-tracking-echocardiography may enhance symptoms identification and improve diagnostic accuracy in the ambulatory settings, especially in subtle cardiac structural and functional abnormalities. Speckle-tracking-echocardiography provides a direct measurement of intrinsic LA myocardial deformation and, although not load independent, LA strain seems to be less dependent on loading conditions and geometric assumptions than traditional parameters with the further advantage to show good feasibility and reproducibility [24, 25]. Moreover, it correlates well with the burden of myocardial fibrosis, i.e. histological marker of chamber stiffness [26, 27].

A “stiff LA syndrome” has been described [11] as clinical consequence of LA dysfunction in the presence of preserved LV function. The clinical usefulness to assess symptoms has been previously investigated in homogeneous study population affected by HFpEF, hypertrophic cardiomyopathy or heart valve disease [28, 29]. However, the coexistence of other conditions, which are hierarchically considered the source of symptoms, make it challenging to understand the additional role of LA. It is possible that the LA chronic exposure to hemodynamic, neuro-endocrinal and metabolic stressors such as those characterizing hypertension, diabetes and CAD favor structural and functional changes that may determine symptoms onset even before macroscopic cardiac structural changes. This concept is in line with the description of a specific “atrial cardiomyopathy” caused primarily by an atrial remodeling process related to external etiological factors directly affecting the LA [30]. From this perspective, atrial cardiomyopathy per se may involved in the risk of stroke before the onset of AF, and as well it may be responsible of neuro-hormonal activation regardless of the hemodynamic status [31]. The pathological changes related to these mechanisms may reflect a spectrum of atrial tissue structural alterations leading to atrial impairment, including myocyte hypertrophy, necrosis, apoptosis and changes in extracellular matrix composition [10, 20] favoring atrial stiffness.

Our results have important clinical implications since it has been recognized that patients with systolic dysfunction and isolated diastolic dysfunction may remain free of HF symptoms [4, 5] and it is still not clear which are the determinants of the transition from the asymptomatic to the symptomatic phase. Moreover, such discrepancy between detected cardiac abnormalities and unrecognized HF symptoms contributes to misdiagnosis of HF [32–34] especially in the context of preserved LVEF. We demonstrated that the presence of atrial myopathy is associated with non-specific symptoms, and it could contribute to their interpretation adding incremental information to the conventional non-invasive measures of cardiac structure and function and comorbidities. The presence of atrial myopathy should raise the suspicion for overt HF and therefore suggest further functional evaluation.

There are several limitations in this study. First, this was a single-center study with a limited number of patients. Second, an erroneous classification of patients as asymptomatic or symptomatic for HF or the presence of non-cardiac sources of symptoms cannot be excluded. Third, measures of NT-pro-BNP plasma levels were not available. Nevertheless, our results suggested the utility of LA measures in an ambulatory setting whereas NT-pro-BNP has been shown to be a marker associated with HF symptoms especially in the acute setting thanks to its high negative predictive value. Of note, it is still not known whether LA functional parameters diagnostic value could apply similarly to natriuretic peptides, i.e. normal LA strain could be used to exclude HF related symptoms. Large cohost studies are needed to investigate LA function thresholds for this purpose.

The echocardiographic estimation of PASP was not included in the analyses since pulmonary hemodynamic alterations were considered as consequence of left chambers remodeling and dysfunction. However, the association between symptoms and LA structural and functional impairment was not affected by introducing PASP values in the analyses (data not shown). Fourth, the diastolic dysfunction grading was not considered in the analyses, since LAVi is one of the four variables considered in diastolic dysfunction stratification [19] and therefore, diastolic dysfunction assessment is strongly dependent on left atrial remodeling. Moreover, the four recommended parameters did not receive an extensive validation in patients with HFpEF and only a modest correlation with invasive filling pressure has been described [35]. Our data showed that LA myopathy was associated with symptomatic status with at least the same predictive value of LA pressure estimated by means of recommended algorithms. Therefore, it could be speculated that the predictability of diastolic dysfunction grading is strongly guided by LA remodeling.

## Conclusions

In ambulatory patients with HF stages A−B and preserved LVEF, measures of LA structure and function are significantly associated with non-specific symptoms, even after accounting for risk factors, comorbidities and measures of cardiac structure and systolic/diastolic function. LA myopathy assessment through LA dilatation and/or reservoir and booster function impairment may add incremental value in the recognition of mild symptoms, which represent the transition from the asymptomatic (stage B) to the symptomatic (stage C) phase of HF. Therefore, a comprehensive LA remodeling evaluation may help clinicians in the appropriate identification of overt HF.

## Supplementary Information

Below is the link to the electronic supplementary material.

Supplementary file1 (DOC 132 kb)

## Data Availability

Available.

## References

[1] Miner B, Tinetti ME, Van Ness PH, Han L, Leo-Summers L, Newman AB, Lee PJ, Vaz Fragoso CA (2016). Dyspnea in community-dwelling older persons: a multifactorial geriatric health condition. J Am Geriatr Soc.

[2] Santos M, Kitzman DW, Matsushita K, Loehr L, Sueta CA, Shah AM (2016). Prognostic importance of dyspnea for cardiovascular outcomes and mortality in persons without prevalent cardiopulmonary disease: the Atherosclerosis Risk in Communities study. PLoS ONE.

[3] Ramalho SHR, Santos M, Claggett B, Matsushita K, Kitzman DW, Loehr L, Solomon SD, Skali H, Shah AM (2019). Association of undifferentiated dyspnea in late life with cardiovascular and noncardiovascular dysfunction a cross-sectional analysis from the ARIC study. JAMA Netw Open.

[4] Benfari G, Miller WL, Antoine C, Rossi A, Lin G, Oh JK, Roger VL, Thapa P, Enriquez-Sarano M (2019). Diastolic determinants of excess mortality in heart failure with reduced ejection fraction. JACC Heart Fail.

[5] Redfield MM, Jacobsen SJ, Burnett JC, Mahoney DW, Bailey KR, Rodeheffer RJ (2003). Burden of systolic and diastolic ventricular dysfunction in the community: appreciating the scope of the heart failure epidemic. JAMA.

[6] McDonagh TA, Morrison CE, Lawrence A, Ford I, Tunstall-Pedoe H, McMurray JJV, Dargie HJ (1997). Symptomatic and asymptomatic left-ventricular systolic dysfunction in an urban population. Lancet.

[7] Pedersen F, Raymond I, Mehlsen J, Atar D, Hildebrandt PR (2005). Prevalence of diastolic dysfunction as a possible cause of dyspnea in the elderly. Am J Med.

[8] Douglas PS (2003). The left atrium: a biomarker of chronic diastolic dysfunction and cardiovascular disease risk. J Am Coll Cardiol.

[9] Inciardi RM, Giugliano RP, Claggett B, Gupta DK, Chandra A, Ruff CT, Antman EM, Mercuri MF, Grosso MA, Braunwald E, Solomon SD, ENGAGE AF-TIMI 48 Investigators (2019). Left atrial structure and function and the risk of death or heart failure in atrial fibrillation. Eur J Heart Fail.

[10] Hoit BD (2014). Left atrial size and function: role in prognosis. J Am Coll Cardiol.

[11] Mehta S, Charbonneau F, Fitchett DH, Marpole DG, Patton R, Sniderman AD (1991). The clinical consequences of a stiff left atrium. Am Heart J.

[12] Mateescu AD, Călin A, Beladan CC, Roşca M, Enache R, Băicuş C, Botezatu S, Ginghină C, Popescu BA (2019). Left atrial dysfunction as an independent correlate of heart failure symptoms in patients with severe aortic stenosis and preserved left ventricular ejection fraction. J Am Soc Echocardiogr.

[13] McKee PA, Castelli WP, McNamara P, Kannel WB (1971). The natural history of congestive heart failure: the Framingham study. N Engl J Med.

[14] Lang RM, Badano LP, Mor-Avi V, Afilalo J, Armstrong A, Ernande L, Flachskampf FA, Foster E, Goldstein SA, Kuznetsova T, Lancellotti P, Muraru D, Picard MH, Rietzschel ER, Rudski L, Spencer KT, Tsang W, Voigt JU (2015). Recommendations for cardiac chamber quantification by echocardiography in adults: an update from the American Society of Echocardiography and the European Association of Cardiovascular Imaging. J Am Soc Echocardiogr.

[15] Zoghbi WA, Adams D, Bonow RO, Enriquez-Sarano M, Grayburn PA, Hahn RT, Han Y, Hung J, Lang RM, Little SH, Shah DJ, Shernan S, Thavendiranathan P, Thomas JD, Weissman NJ (2017). Recommendations for noninvasive evaluation of native valvular regurgitation a report from the american society of echocardiography developed in collaboration with the society for cardiovascular magnetic resonance. J Am Soc Echocardiogr.

[16] Pernigo M, Benfari G, Geremia G, Noni M, Borio G, Mazzali G, Zamboni M, Onorati F, Faggian G, Vassanelli C, Rossi A (2017). Atrial function as an independent predictor of postoperative atrial fibrillation in patients undergoing aortic valve surgery for severe aortic stenosis. J Am Soc Echocardiogr.

[17] Pathan F, D’Elia N, Nolan MT, Marwick TH, Negishi K (2016). Normal ranges of left atrial strain by speckle-tracking echocardiography: a systematic review and meta-analysis. J Am Soc Echocardiogr.

[18] Cuzick J (1985). A Wilcoxon-type test for trend. Stat Med.

[19] Nagueh SF, Smiseth OA, Appleton CP, Byrd BF, Dokainish H, Edvardsen T, Flachskampf FA, Gillebert TC, Klein AL, Lancellotti P, Marino P, Oh JK, Popescu BA, Waggoner AD (2016). Recommendations for the evaluation of left ventricular diastolic function by echocardiography: an update from the American Society of Echocardiography and the European Association of Cardiovascular Imaging. J Am Soc Echocardiogr.

[20] Rossi A, Triposkiadis F, Solomon SD, Pieske B, Butler J (2014). Left atrium in heart failure with preserved ejection fraction structure, function, and significance. Circ Heart Fail.

[21] Khan MS, Memon MM, Murad MH, Vaduganathan M, Greene SJ, Hall M, Triposkiadis F, Lam CSP, Shah AM, Butler J, Shah SJ (2020). Left atrial function in heart failure with preserved ejection fraction: a systematic review and meta-analysis. Eur J Heart Fail.

[22] Vonk Noordegraaf A, Westerhof BE, Westerhof N (2017). The relationship between the right ventricle and its load in pulmonary hypertension. J Am Coll Cardiol.

[23] Inciardi RM, Rossi A, Bergamini C, Benfari G, Maffeis C, Greco C, Drago A, Guazzi M, Ribichini FL, Cicoira M (2020). Mitral Regurgitation, Left Atrial Structural and Functional Remodeling and the Effect on Pulmonary Hemodynamics. Eur J Heart Fail.

[24] Zhang Q, Yip GW, Yu CM (2008). Approaching regional left atrial function by tissue Doppler velocity and strain imaging. Europace.

[25] Cameli M, Caputo M, Mondillo S, Ballo P, Palmerini E, Lisi M, Marino E, Galderisi M (2009). Feasibility and reference values of left atrial longitudinal strain imaging by two-dimensional speckle tracking. Cardiovasc Ultrasound.

[26] Cameli M, Lisi M, Righini FM, Massoni A, Natali BM, Focardi M, Tacchini D, Geyer A, Curci V, Di Tommaso C, Lisi G, Maccherini M, Chiavarelli M, Massetti M, Tanganelli P, Mondillo S (2013). Usefulness of atrial deformation analysis to predict left atrial fibrosis and endocardial thickness in patients undergoing mitral valve operations for severe mitral regurgitation secondary to mitral valve prolapse. Am J Cardiol.

[27] Benfari G, Noni M, Onorati F, Cerrito LF, Pernigo M, Vinco G, Cameli M, Mandoli GE, Borio G, Geremia G, Zivelonghi C, Abbasciano R, Mazzali G, Zamboni M, Faggian G, Rossi A, Ribichini FL (2019). Effects of aortic valve replacement on left ventricular diastolic function in patients with aortic valve stenosis. Am J Cardiol.

[28] Sanchis L, Gabrielli L, Andrea R, Falces C, Duchateau N, Perez-Villa F, Bijnens B, Sitges M (2015). Left atrial dysfunction relates to symptom onset in patients with heart failure and preserved left ventricular ejection fraction. Eur Heart J Cardiovasc Imaging.

[29] Roşca M, Popescu BA, Beladan CC, Călin A, Muraru D, Popa EC, Lancellotti P, Enache R, Coman IM, Jurcuţ R, Ghionea M, Ginghină C (2010). Left atrial dysfunction as a correlate of heart failure symptoms in hypertrophic cardiomyopathy. J Am Soc Echocardiogr.

[30] Guichard JB, Nattel S (2017). Atrial cardiomyopathy: a useful notion in cardiac disease management or a passing fad?. J Am Coll Cardiol.

[31] Rossi A, Enriquez-Sarano M, Burnett JC, Lerman A, Abel MD, Seward JB (2000). Natriuretic peptide levels in atrial fibrillation a prospective hormonal and doppler-echocardiographic study. J Am Coll Cardiol.

[32] Valk MJ, Mosterd A, Broekhuizen BDL, Zuithoff NPA, Landman MAJ, Hoes AW, Rutten FH (2016). Overdiagnosis of heart failure in primary care: a cross-sectional study. Br J Gen Pract.

[33] van Riet EE, Hoes AW, Limburg A, Landman MA, van der Hoeven H, Rutten FH (2014). Prevalence of unrecognized heart failure in older persons with shortness of breath on exertion. Eur J Heart Fail.

[34] Rutten FH, Cramer MJ, Grobbee DE, Sachs APE, Kirkels JH, Lammers JWJ, Hoes AW (2005). Unrecognized heart failure in elderly patients with stable chronic obstructive pulmonary disease. Eur Heart J.

[35] Nauta JF, Hummel YM, van der Meer P, Lam CSP, Voors AA, van Melle JP (2018). Correlation with invasive left ventricular filling pressures and prognostic relevance of the echocardiographic diastolic parameters used in the 2016 ESC heart failure giudelines and in the 2016 ASE/EACVI recommendations: a systematic review in patients with heart failure with preserved ejection fraction. Eur J Heart Fail.

