# Supplementary material for: Determinants of exercise intolerance symptoms considered non-specific for heart failure in patients with stage A and B: role of the left atrium in the transition phase to overt heart failure
Source: Int J Cardiovasc Imaging. 2021 Aug 30;38(1):103–12. doi: 10.1007/s10554-021-02375-0 (PMC8818638; doi:10.1007/s10554-021-02375-0)

**SUPPLEMETAL MATERIALS**

**Supplementary Figure 1.** Receiver operating characteristic (ROC) curve evaluating the performance of ~~analysis for symptoms status predictive value of estimated grade of~~ guidelines directed estimated left atrial (LA) pressure (normal, elevated with grade II diastolic dysfunction, elevated with grade III diastolic dysfunction) versus four stages of LA myopathy (defined by the presence of left atrial enlargement and/or impaired function, see text for details) in predicting the symptomatic status. LA pressure was indeterminable in 23 (12%) patients.

The continuous line represents the ROC curve for LA myopathy, the dotted line represents the ROC curve for LA pressure degree.

LA myopathy AUC: 0.83 versus LA pressure AUC: 0.68 (p<0.05)**.**

**Supplementary Table 1.** Diastolic dysfunction grades according to guidelines recommended algorithms.

|  | Mean ± SD | | P value | P value  Adjusted for Model 1 |
| --- | --- | --- | --- | --- |
|  | Asymptomatic n=133 (72%) | Symptomatic n=52 (28%) |
| Diastolic dysfunction, n (%)  Grade I  Grade II  Grade III  Indeterminate | 65(48)  48(36)  6(4)  2(1)  9(7) | 43(83)  22(42)  19(36)  2(4)  0(0) | <0.0001 | 0.01 |

**Supplementary Table 2. Measures of LA structure and function according to LA structure (LAVi >34 ml/m^2^) and function (PALS <38%).**

|  | Normal LA structure (n=99) | Altered LA structure (n=86) | P value | Normal LA function (n=66) | Reduced LA function (n=119) | P value |
| --- | --- | --- | --- | --- | --- | --- |
| LAVi, ml/m^2^ | 25.7±5.0 [15-34] | 47.8±13.1 [35-108] | <0.0001 | 26.7±7.2 [15-53] | 40.6±15.1 [15-108] | <0.0001 |
| LA PALS, % | 38.8±9.3 [22-60] | 27.1±9.3 [10-55] | <0.0001 | 45.5±6.1 [38-60] | 26.8±6.6 [10-37] | <0.0001 |
| LA PACS, % | 18.0±6.2 [5-34] | 12.3±5.4 [0-26] | <0.0001 | 20.2±5.5 [8-34] | 12.7±5.4 [0-26] | <0.0001 |
| LA CS, % | 21.2±7.4 [7-40] | 12.3±6.5 [5-35] | <0.0001 | 25.5±6.1 [8-40] | 14.2±5.0 [5-26] | <0.0001 |

Abbreviations: LAVi: left atrial volume index; LA PALS: LA peak atrial longitudinal strain; LA PACS: LA peak atrial contraction strain; LA CS: conduit strain.

**Supplementary Table 3. Predictive value for symptomatic status of three grades of guidelines directed estimated left atrial pressure (normal; elevated with grade II diastolic dysfunction; elevated with grade III diastolic dysfunction) versus four stages of atrial myopathy defined by the presence of left atrial enlargement and/or impaired function (see text for details). Left atrial pressure was indeterminable in 23 (12%) patients.**

|  | Unadjusted | | Model 1 | | Model 2 | |
| --- | --- | --- | --- | --- | --- | --- |
|  | OR, 95%CI | P value | OR, 95%CI | P value | OR, 95%CI | P value |
| LAP | 5.9 (2.6-13.4) | <0.0001 | 4.3 (1.3-14.5) | 0.01 | 3.1 (1.2-7.9) | 0.01 |
| LAM 4 groups | 3.1 (2.1-4.5) | <0.0001 | 3.7 (1.7-6.6) | <0.0001 | 2.5 (1.5-4.1) | <0.0001 |

OR, Odds ratio; CI, confidence interval. Model 1: adjusted for Age, Sex, BMI, Hypertension, eGFR, history of coronary artery disease, total cholesterol, diabetes, COPD; Model 2: adjusted for S-TDI, LV mass-index, E/E', MR-ERO**.**

Abbreviations: LAM: left atrial myopathy; LAP: left atrial pressure.

**Supplementary Table 4.** Association between symptomatic status and measures of LA structure and function in patients without history of atrial fibrillation

|  | **Unadjusted** | | **Model 1** | | **Model 2** | |
| --- | --- | --- | --- | --- | --- | --- |
|  | **OR, 95%CI** | **p-value** | **OR, 95%CI** | **p-value** | **OR, 95%CI** | **p-value** |
| **LAVi, ml/m2** | **1.43(1.23-1.67)** | **<0.0001** | **1.80(1.30-2.52)** | **<0.0001** | **1.39(1.08-1.78)** | **0.009** |
| **LA PALS, %** | **1.57(1.28-1.93)** | **<0.0001** | **1.44(1.06-1.95)** | **0.01** | **1.24(0.97-1.59)** | **0.09** |
| **LA PACS, %** | **1.77(1.30-2.42)** | **<0.0001** | **2.08(1.25-3.46)** | **0.005** | **1.52(1.03-2.23)** | **0.03** |
| **LA CS, %** | **1.59(1.21-2.08)** | **<0.0001** | **1.17(0.78-1.75)** | **0.40** | **1.03(0.73-1.47)** | **0.80** |

OR, Odds ratio; CI, confidence interval; OR is expressed per 5-unit change in each measure of LAVi (increase) and LA function (decrease). Model 1: adjusted for Age, Sex, BMI, Hypertension, eGFR, history of coronary artery disease, total cholesterol, diabetes, COPD; Model 2: adjusted for S-TDI, LV mass-index, E/E', MR-ERO**.**

Abbreviations: LAVi: left atrial volume index; LA PALS: LA peak atrial longitudinal strain; LA PACS: LA peak atrial contraction strain; LA CS: conduit strain.

**Supplementary Figure 1.**


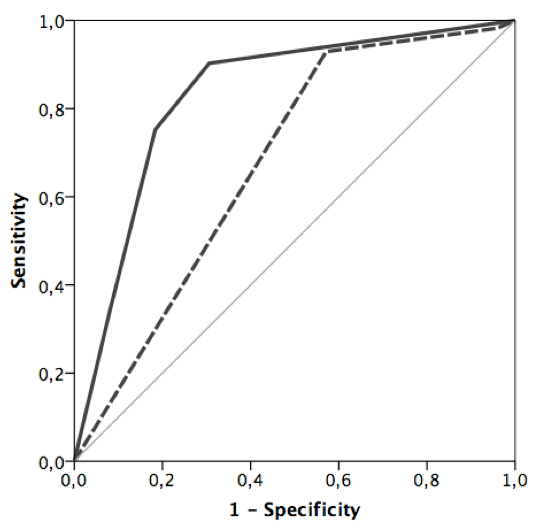

Supplement: Supplementary file 1 — Supplementary file1 (DOC 132 kb) [file 10554_2021_2375_MOESM1_ESM.doc]
